# Supplementary material for: Personalized Assessment of Normal Tissue Radiosensitivity via Transcriptome Response to Photon, Proton and Carbon Irradiation in Patient-Derived Human Intestinal Organoids
Source: Cancers (Basel). 2020 Feb 18;12(2):469. doi: 10.3390/cancers12020469 (PMC7072449; doi:10.3390/cancers12020469)
Supplement: Supplementary file 1 [file cancers-12-00469-s001.zip › cancers-706804-supplementary/Supplementary Figures Nowrouzi et al_HIO_PRO_130120.pdf]

Supplementary Figures

Personalized assessment of normal tissue radiosensitivity via transcriptome response to photon, proton and carbon irradiation in patient derived human intestinal organoids

Ali Nowrouzi[1,2,3,4*], Mathieu G. Sertorio[6], Mahdi Akbarpour[1,2,3,4], Maximillian Knoll[1,2,3,4], Damir Krunic[5], Matthew Kuhar[7], Christian Schwager[1,2,3,4], Stephan Brons[3], Jürgen Debus[1,2,3,4], Susanne I. Wells[6], James M. Wells[7,8,9] and Amir Abdollahi[1,2,3,4]

[1] Heidelberg Ion-Beam Therapy Center (HIT), Department of Radiation Oncology, Heidelberg University Hospital (UKHD), Heidelberg, Germany
[2] German Cancer Consortium (DKTK) Core Center, Clinical Cooperation Units (CCU) Translational Radiation Oncology and Radiation Oncology, National Center for Tumor Diseases (NCT), German Cancer Research Center (DKFZ), Heidelberg, Germany
[3] Heidelberg Institute of Radiation Oncology (HIRO), National Center for Radiation Research in Oncology (NCRO), German Cancer Research Center (DKFZ) and Heidelberg University Hospital (UKHD), Heidelberg, Germany
[4] Division of Molecular and Translational Radiation Oncology, Heidelberg Medical Faculty (HDMF), Heidelberg University, Heidelberg, Germany
[5] Light Microscopy Facility, German Cancer Research Center, Heidelberg 69120, Germany
[6] Cancer and Blood Diseases Institute, Cincinnati Children’s Hospital Medical Center, Cincinnati, OH, USA
[7] Division of Developmental Biology, Cincinnati Children's Hospital Medical Center, Cincinnati, Ohio, USA
[8] Division of Endocrinology, Cincinnati Children's Hospital Medical Center, Cincinnati, Ohio, USA
[9] Center for Stem Cell and Organoid Medicine, Cincinnati Children's Hospital Medical Center, Cincinnati, Ohio, USA

* Correspondence: a.nowrouzi@dkfz.de; Tel.: +49-6221-56-34638 (Heidelberg, Germany)

FigS 1

a.

Proton compared to Photon

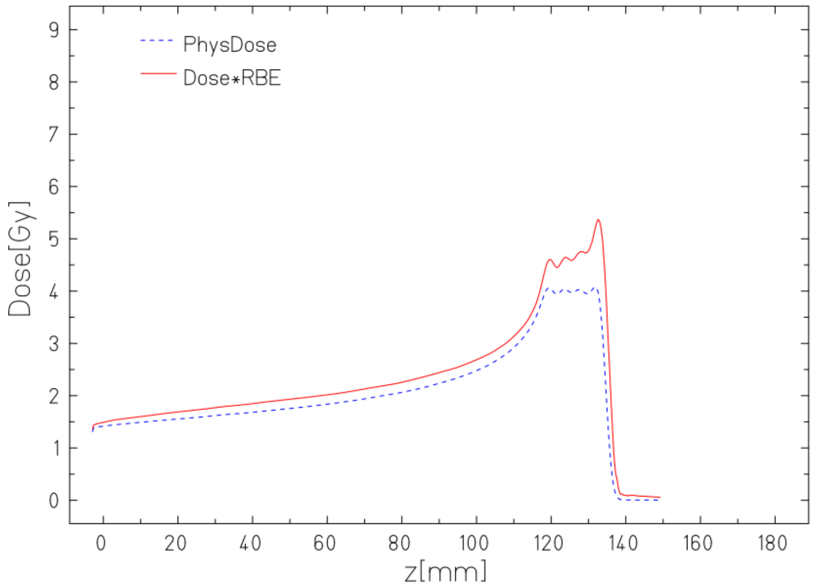

b.

Carbon compared to Photon

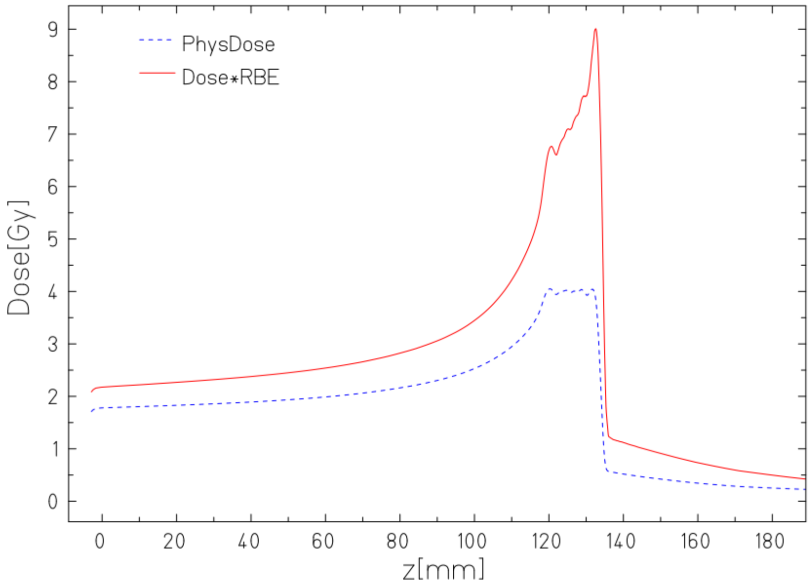

FigS 2

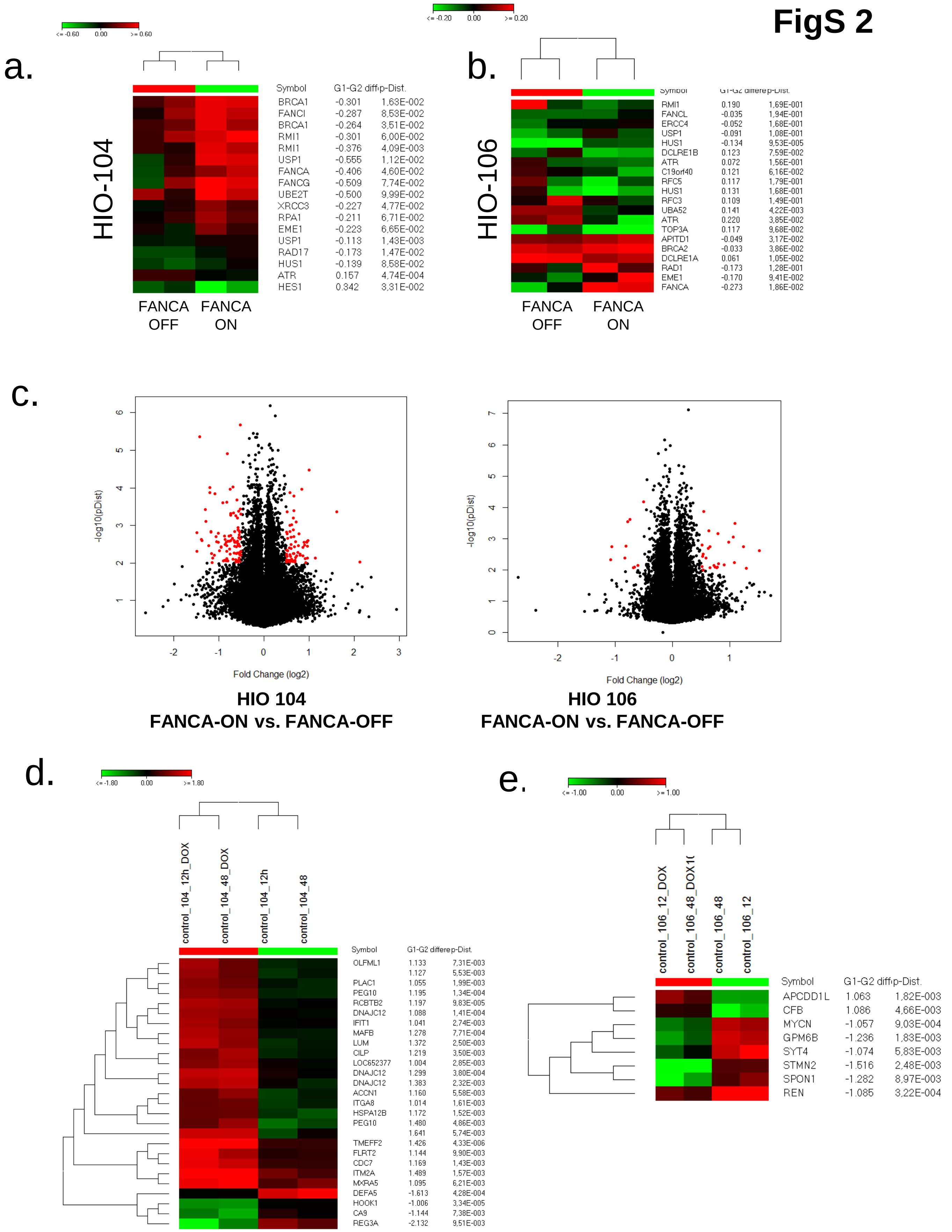

a.
<= -0.60 0.00 >= 0.60
HIO-104
Symbol G1-G2 diff p-Dist.
BRCA1 -0.301 1,63E-002
FANCI -0.287 8,53E-002
BRCA1 -0.264 3,51E-002
RMI1 -0.301 6,00E-002
RMI1 -0.376 4,09E-003
USP1 -0.555 1,12E-002
FANCA -0.406 4,60E-002
FANCG -0.509 7,74E-002
UBE2T -0.500 9,99E-002
XRCC3 -0.227 4,77E-002
RPA1 -0.211 6,71E-002
EME1 -0.223 6,65E-002
USP1 -0.113 1,43E-003
RAD17 -0.173 1,47E-002
HUS1 -0.139 8,58E-002
ATR 0.157 4,74E-004
HES1 0.342 3,31E-002
FANCA OFF
FANCA ON
b.
<= -0.20 0.00 >= 0.20
HIO-106
RMI1 0.190 1,69E-001
FANCL -0.035 1,94E-001
ERCC4 -0.052 1,68E-001
USP1 -0.091 1,08E-001
HUS1 -0.134 9,53E-005
DCLRE1B 0.123 7,59E-002
ATR 0.072 1,56E-001
C19orf40 0.121 6,16E-002
RFC5 0.117 1,79E-001
HUS1 0.131 1,68E-001
RFC3 0.109 1,49E-001
UBA52 0.141 4,22E-003
ATR 0.220 3,85E-002
TOP3A 0.117 9,68E-002
APITD1 -0.049 3,17E-002
BRCA2 -0.033 3,86E-002
DCLRE1A 0.061 1,05E-002
RAD1 -0.173 1,28E-001
EME1 -0.170 9,41E-002
FANCA -0.273 1,86E-002
FANCA OFF
FANCA ON
c.
-log10(pDist)
Fold Change (log2)
HIO 104
FANCA-ON vs. FANCA-OFF
HIO 106
FANCA-ON vs. FANCA-OFF
d.
<= -1.80 0.00 >= 1.80
control_104_12h_DOX
control_104_48_DOX
control_104_12h
control_104_48
Symbol G1-G2 differe p-Dist.
OLFML1 1.133 7,31E-003
1.127 5,53E-003
PLAC1 1.055 1,99E-003
PEG10 1.195 1,34E-004
RCBTB2 1.197 9,83E-005
DNAJC12 1.088 1,41E-004
IFIT1 1.041 2,74E-003
MAFB 1.278 7,71E-004
LUM 1.372 2,50E-003
CILP 1.219 3,50E-003
LOC652377 1.004 2,85E-003
DNAJC12 1.299 3,80E-004
DNAJC12 1.383 2,32E-003
ACCN1 1.160 5,58E-003
ITGA8 1.014 1,61E-003
HSPA12B 1.172 1,52E-003
PEG10 1.480 4,86E-003
1.641 5,74E-003
TMEFF2 1.426 4,33E-006
FLRT2 1.144 9,90E-003
CDC7 1.169 1,43E-003
ITM2A 1.489 1,57E-003
MXRA5 1.095 6,21E-003
DEFA5 -1.613 4,28E-004
HOOK1 -1.006 3,34E-005
CA9 -1.144 7,38E-003
REG3A -2.132 9,51E-003
e.
<= -1.00 0.00 >= 1.00
control_106_12_DOX
control_106_48_DOX
control_106_48
control_106_12
Symbol G1-G2 diff p-Dist.
APCDD1L 1.063 1,82E-003
CFB 1.086 4,66E-003
MYCN -1.057 9,03E-004
GPM6B -1.236 1,83E-003
SYT4 -1.074 5,83E-003
STMN2 -1.516 2,48E-003
SPON1 -1.282 8,97E-003
REN -1.085 3,22E-004

FigS 3

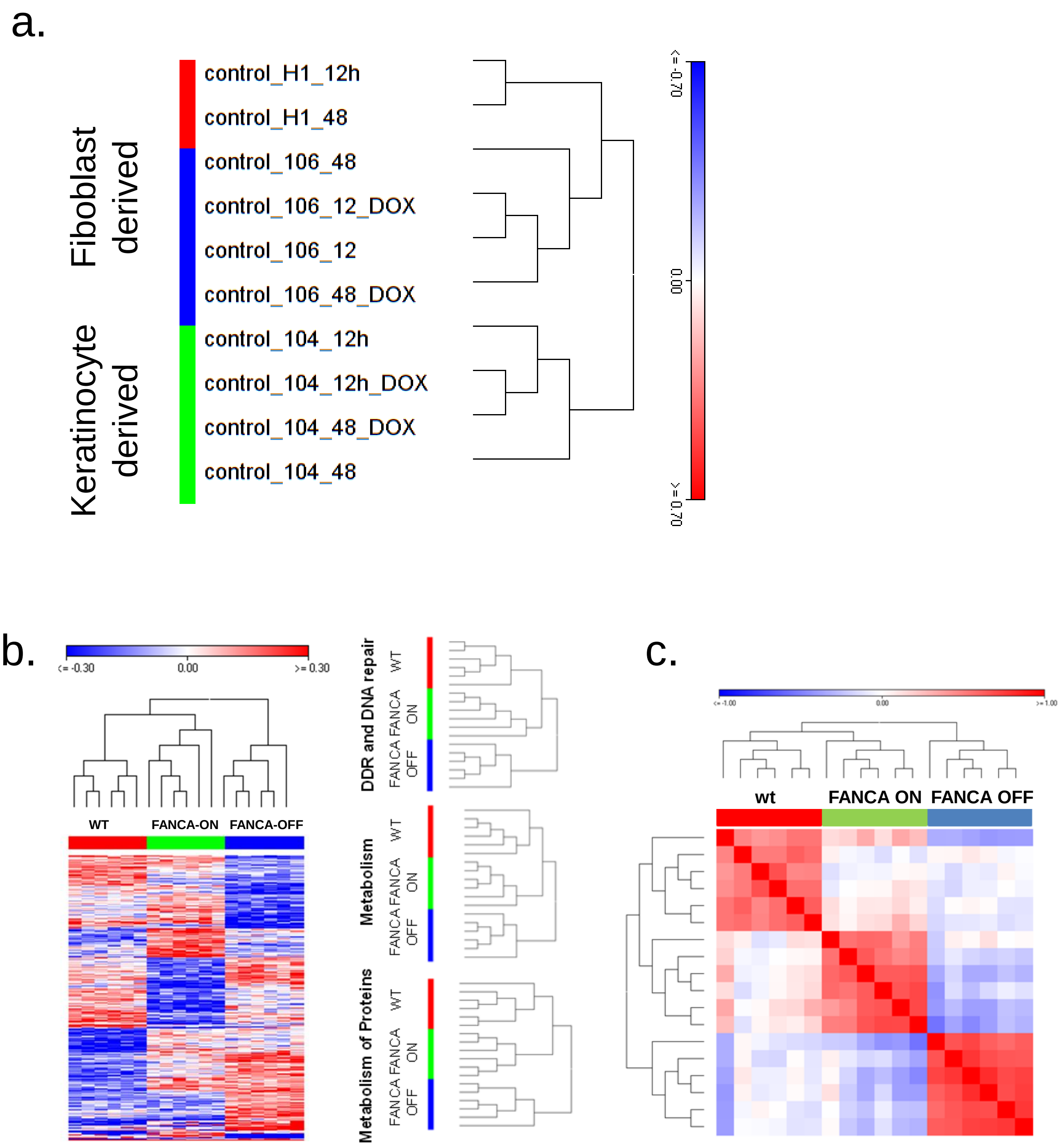

a.
Fiboblast derived
Keratinocyte derived
control_H1_12h
control_H1_48
control_106_48
control_106_12_DOX
control_106_12
control_106_48_DOX
control_104_12h
control_104_12h_DOX
control_104_48_DOX
control_104_48
<= -0.70
0.00
>= 0.70
b.
<= -0.30
0.00
>= 0.30
WT
FANCA-ON
FANCA-OFF
DDR and DNA repair
Metabolism
Metabolism of Proteins
WT
FANCA ON
FANCA OFF
c.
<= -1.00
0.00
>= 1.00
wt
FANCA ON
FANCA OFF

FigS 4

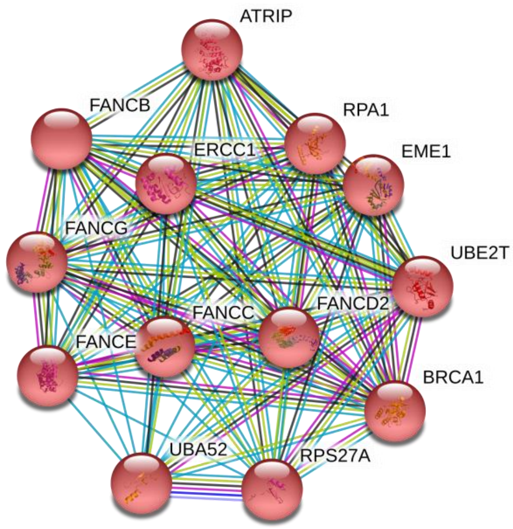

Fanconi Anemia Pathway

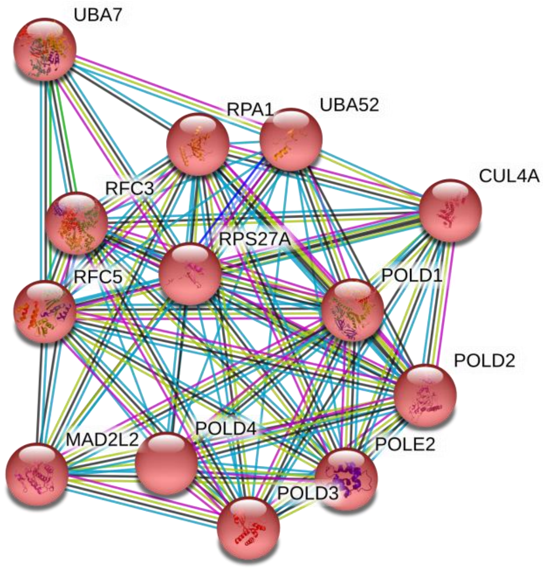

Translesion Synthesis

FigS 5

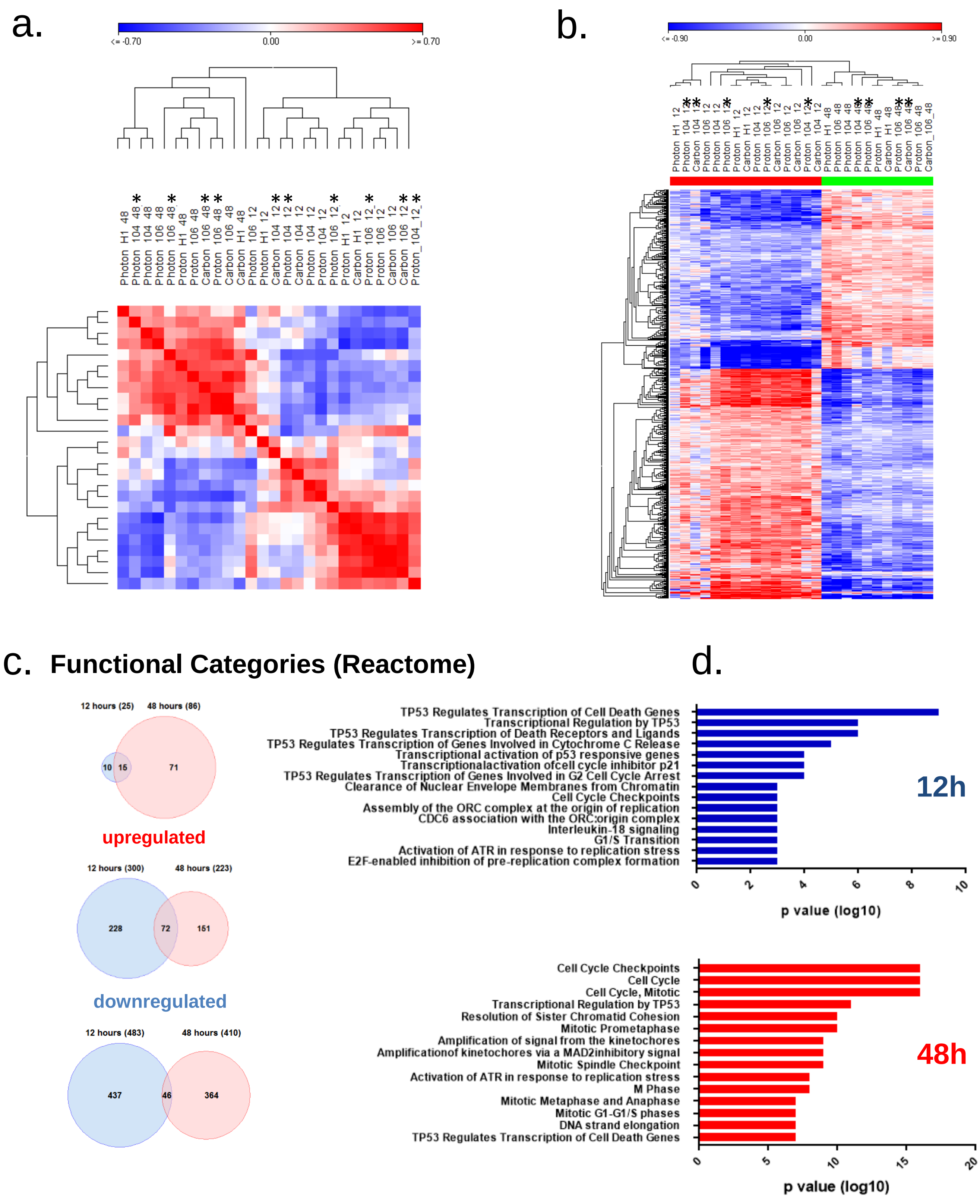

a.
<= -0.70
0.00
>= 0.70
Photon H1 48
Photon 104 48
Photon 104 48
Photon 106 48
Photon 106 48
Proton H1 48
Proton 106 48
Carbon 106 48
Proton 106 48
Carbon 106 48
Carbon H1 48
Photon 106 12
Photon H1 12
Carbon 104 12
Photon 104 12
Carbon 104 12
Proton 104 12
Photon 106 12
Proton H1 12
Carbon H1 12
Proton 106 12
Proton 106 12
Carbon 106 12
Proton_104_12
b.
<= -0.90
0.00
>= 0.90
c.
Functional Categories (Reactome)
12 hours (25)
48 hours (86)
10
15
71
upregulated
12 hours (300)
48 hours (223)
228
72
151
downregulated
12 hours (483)
48 hours (410)
437
46
364
d.
TP53 Regulates Transcription of Cell Death Genes
Transcriptional Regulation by TP53
TP53 Regulates Transcription of Death Receptors and Ligands
TP53 Regulates Transcription of Genes Involved in Cytochrome C Release
Transcriptional activation of p53 responsive genes
Transcriptionalactivation ofcell cycle inhibitor p21
TP53 Regulates Transcription of Genes Involved in G2 Cell Cycle Arrest
Clearance of Nuclear Envelope Membranes from Chromatin
Cell Cycle Checkpoints
Assembly of the ORC complex at the origin of replication
CDC6 association with the ORC:origin complex
Interleukin-18 signaling
G1/S Transition
Activation of ATR in response to replication stress
E2F-enabled inhibition of pre-replication complex formation
12h
0
2
4
6
8
10
p value (log10)
Cell Cycle Checkpoints
Cell Cycle
Cell Cycle, Mitotic
Transcriptional Regulation by TP53
Resolution of Sister Chromatid Cohesion
Mitotic Prometaphase
Amplification of signal from the kinetochores
Amplificationof kinetochores via a MAD2inhibitory signal
Mitotic Spindle Checkpoint
Activation of ATR in response to replication stress
M Phase
Mitotic Metaphase and Anaphase
Mitotic G1-G1/S phases
DNA strand elongation
TP53 Regulates Transcription of Cell Death Genes
48h
0
5
10
15
20
p value (log10)

FigS 6

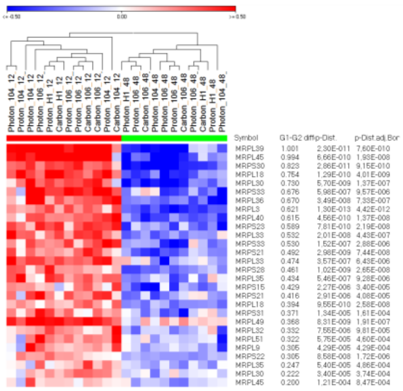

Mitochondrial Translation

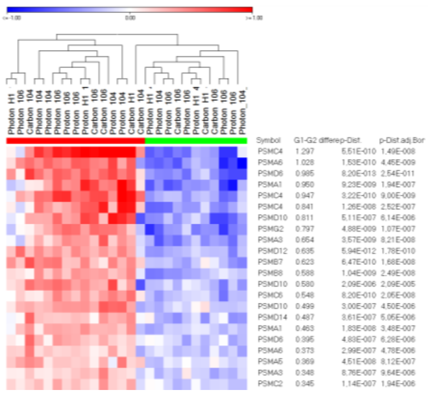

Ubiquitin Mediated Proteasome Degradation

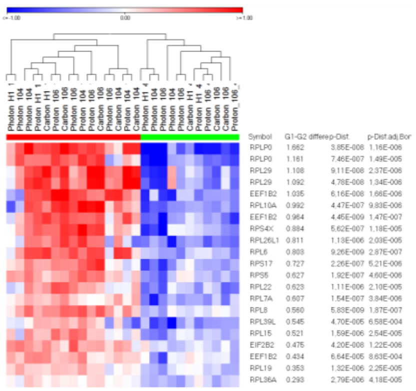

Eukaryotic Translation

FigS 7

a.

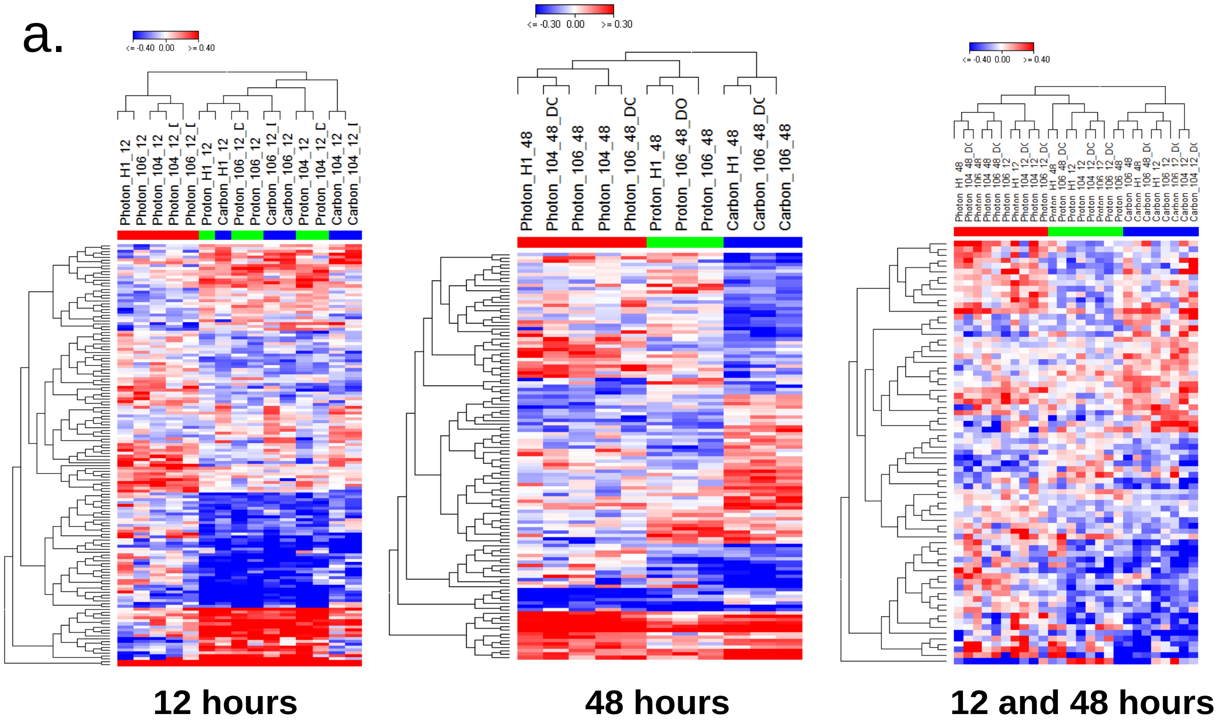

<= -0.40 0.00 >= 0.40
<= -0.30 0.00 >= 0.30
Photon_H1_48
Photon_104_48_DC
Photon_106_48
Photon_104_48
Photon_106_48_DC
Proton_H1_48
Proton_106_48_DO
Proton_106_48
Carbon_H1_48
Carbon_106_48_DC
Carbon_106_48
12 hours
48 hours
12 and 48 hours

b.

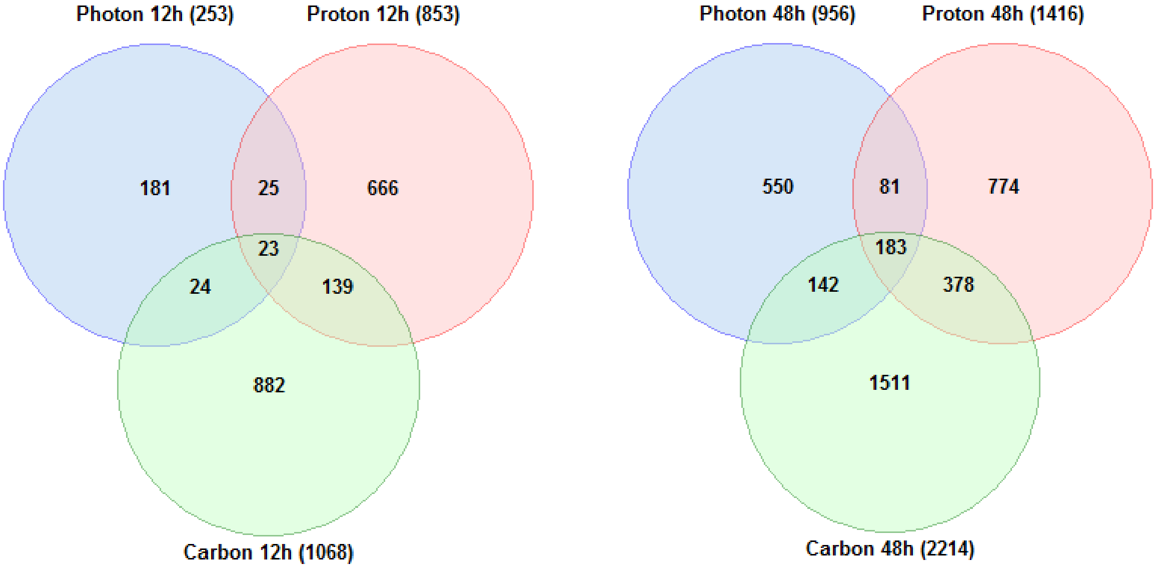

Photon 12h (253)
Proton 12h (853)
181
25
666
23
24
139
882
Carbon 12h (1068)
Photon 48h (956)
Proton 48h (1416)
550
81
774
183
142
378
1511
Carbon 48h (2214)

FigS 8

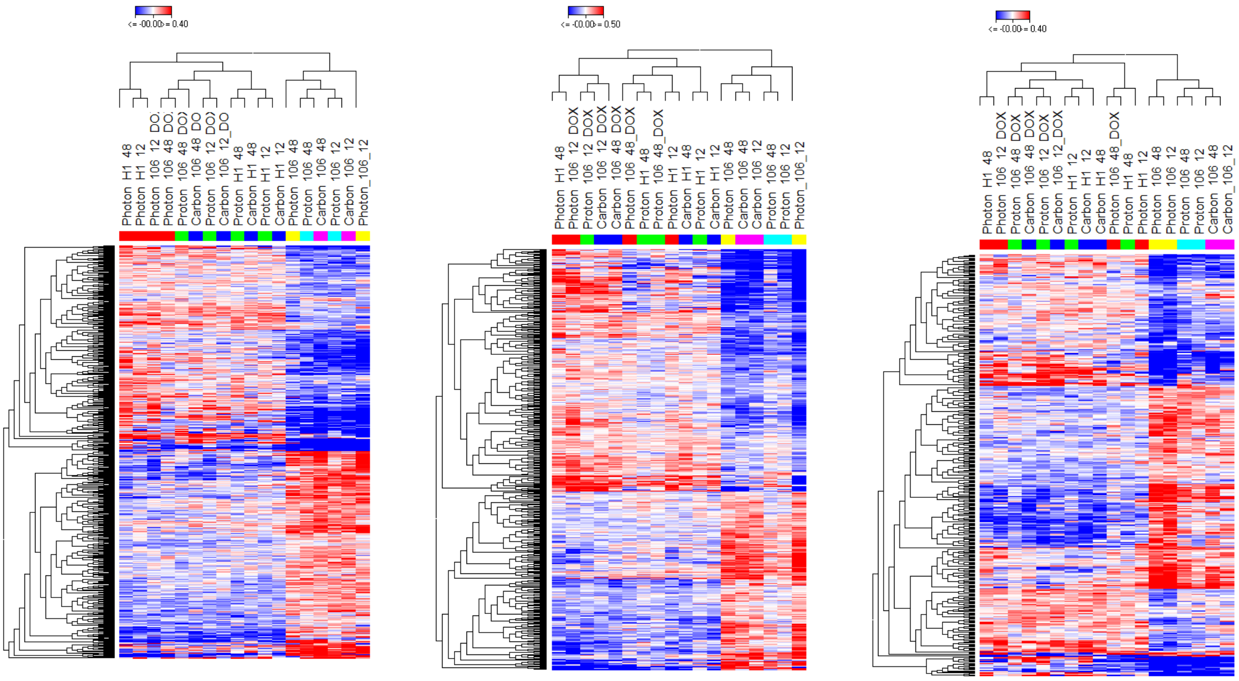

FA RQ FA and RQ
